# Probing the PI3K/Akt/mTor pathway using ^31^P-NMR spectroscopy: routes to glycogen synthase kinase 3

**DOI:** 10.1038/srep36544

**Published:** 2016-11-04

**Authors:** Su M. Phyu, Chih-Chung Tseng, Ian N. Fleming, Tim A. D. Smith

**Affiliations:** 1 School of Medicine, Medical Sciences and Nutrition, University of Aberdeen, Foresterhill, Aberdeen AB25 2ZD, United Kingdom.

## Abstract

Akt is an intracellular signalling pathway that serves as an essential link between cell surface receptors and cellular processes including proliferation, development and survival. The pathway has many downstream targets including glycogen synthase kinase3 which is a major regulatory kinase for cell cycle transit as well as controlling glycogen synthase activity. The Akt pathway is frequently up-regulated in cancer due to overexpression of receptors such as the epidermal growth factor receptor, or mutation of signalling pathway kinases resulting in inappropriate survival and proliferation. Consequently anticancer drugs have been developed that target this pathway. MDA-MB-468 breast and HCT8 colorectal cancer cells were treated with inhibitors including LY294002, MK2206, rapamycin, AZD8055 targeting key kinases in/associated with Akt pathway and the consistency of changes in ^31^P-NMR-detecatable metabolite content of tumour cells was examined. Treatment with the Akt inhibitor MK2206 reduced phosphocholine levels in MDA-MB-468 cells. Treatment with either the phosphoinositide-3-kinase inhibitor, LY294002 and pan-mTOR inhibitor, AZD8055 but not pan-Akt inhibitor MK2206 increased uridine-5′-diphosphate-hexose cell content which was suppressed by co-treatment with glycogen synthase kinase 3 inhibitor SB216763. This suggests that there is an Akt-independent link between phosphoinositol-3-kinase and glycogen synthase kinase3 and demonstrates the potential of ^31^P-NMR to probe intracellular signalling pathways.

The PI3K/Akt/mTOR signalling pathway is activated by several tyrosine kinase receptors including the insulin receptor and the human epithelial receptor family (HER) which includes EGFR^1^. Consequently the PI3K/Akt/mTOR pathway plays an important role in the regulation of many aspects of cell function including metabolism, proliferation, protein synthesis and survival^2^. Cell survival is mediated by activated Akt (phosphorylated Akt) by inhibition of several steps in apoptosis.

Cancer is characterised by uncontrolled proliferation and inappropriate cell survival^3^ and these processes are commonly enhanced in tumours by up-regulation of the PI3K/Akt/mTOR pathway frequently as a result of over-expression of upstream receptors or mutations in components of the pathway or both. Therefore PI3K/Akt/mTOR pathway inhibitors are undergoing clinical trials for cancer treatment^4^.

PI3K catalyses the formation of phosphatidylinositol 3,4-bisphosphate and phosphatidylinositol 3,4,5-trisphosphate (PIP3) which activates many signalling proteins including Akt, phosphatidylinositide-dependent kinase 1(PDK1) and protein kinases A and C. Conversely the phosphatase, PTEN (phosphatase and tensin homolog), inactivates PIP3 by catalysing it’s dephosphorylation to phosphatidylinositol-(4,5)-bisphosphate (PIP2) so down-regulating the activity of these proteins. About 6% and 10% of breast and colorectal cancers (CRC) cancers respectively carry PTEN inactivating mutations^5^. Whilst activating mutations in PI3KCA (phosphatidylinositol-4,5-bisphosphate-3-kinase catalytic subunit alpha) are found in >25% breast tumours and about15% of CRC. These mutations promote tumorigenesis^6^,^7^ and resistance to endocrine, radiotherapy and chemotherapy^8^,^9^ through PI3K/Akt pathway activation. The Akt pathway also controls glucose metabolism^10^ which is an important source of fatty acids for phospholipid synthesis^11^.

Glycogen synthase kinase (GSK), a target of Akt, was originally identified as a kinase that phosphorylates and inactivates glycogen synthase (the final enzyme involved in glycogen synthesis)^12^ but it was later established that GSK has important regulatory roles in several cell functions^13^. GSK exerts a negative regulatory effect on the G_1_ cyclins, cyclin D and E and the transcription factors c-jun and c-myc that are crucial to G_1_ to S transition^14^. In addition to the direct effects of Akt on the regulation of apoptosis and proliferation, pAkt drives the cell cycle by phosphorylating and inactivating (GSK) isoforms resulting in inhibition of its negative regulatory effect on cell cycle progression.

Although the relationship between PI3K and Akt is well established, the links with other signalling molecules down-stream of PI3K is less well characterised. It has been shown that GSK can be controlled by PI3K via PKA which physically complexes with GSK^15^. Recently a further Akt-independent link between PI3K and GSK3 has been identified^16^.

^31^P-NMR spectroscopy is a useful technique for monitoring phospholipid metabolite levels in tissues/cells and extracts and is potentially clinically translatable for therapy response monitoring in patients. Metabolites present in the ^31^P-NMR spectrum from cancer cells include^17^,^18^,^19^ the product of choline kinase (CK) and phospholipase C (PLC), phosphocholine (PCho) and the product of Phospholipase A2 (PLA2) glycerophosphocholine (GPC) from the breakdown of phosphatidylcholine. Studies^20^,^21^,^22^,^23^ have established that the activity of anabolic (CK) and catabolic (PLA2, PLC and PLD) phospholipid enzymes are regulated by the Akt/mTor pathway. Thus the expression and activity of choline kinase are controlled by a complex of which PI3K is a part^20^,^21^. Recent work has demonstrated that protein levels of PLA2 are regulated by Akt which inhibits PLA2 degradation^22^. A further peak evident in ^31^P-NMR spectra of some tumours is the UDP-hexoses peak which includes resonances from UDP-glucose which is converted to glycogen by glycogen synthase.

Several *in-vitro* and *in-vivo* studies^17^,^24^,^25^,^26^,^27^ have applied ^31^P-NMR to measure phospholipid metabolite levels in tumour/cells responding to drugs targeting the PI3K/Akt/mTOR but the findings have not demonstrated a clear consensus especially with respect to their effects on PCho content. Since metabolites may serve as prognostic or predictive biomarkers, we aimed to explore the effect of drugs inhibiting several components of PI3K signalling (see Fig. 1) on metabolite levels in breast cancer cells *in vitro* to determine whether or not metabolic changes detectable using ^31^P-NMR spectroscopy robustly reflect Akt pathway inhibition. Measurements were carried out on extracts from cells treated for 24 h as metabolic changes are observed within hours of treatment of cancer cells with Akt pathway inhibitors^10^ and after 72 h of treatment to determine the consistency of changes detectable after 24 h.

## Materials and Methods

### Materials

The breast and colorectal cancer cell lines, MDA-MB-468 and HCT8 respectively, were purchased from American Type Cell Culture Collection (LGC Standards UK) with authentication. The cell lines constitutively overexpress Akt due to mutant PTEN (MDA-MB-468)^28^ or PI3K (HCT-8)^29^ providing suitable models for exploring Akt inhibition. Unless stated otherwise the chemicals used were purchased from Sigma-Aldrich chemical company. Inhibitors used in this study were LY294002, GSK2334470, H89, Staurosporine, MK2206, rapamycin and AZD8055 targeting PI3K, PDK1, PKA, PKC, Akt, mTOR in mTORC1 and mTOR in mTORC1&2 respectively and SB216763 targeting GSK3. They were all obtained from Cambridge Biosciences (Cambridge, UK). Akt (9272), and p-Akt (Ser473) (9271) antibodies were from Cell signalling technology, GSKβ (sc-9166) and p-GSKβ (ser-9) (sc-11757) antibodies were from Santa Cruz biotechnology. Β-Actin antibody (clone AC-15) was from Sigma-Aldrich. Universally labelled (all Cs) [^14^C-U]glucose (9.25–13.3 GBq/mmol) was obtained from Perkin-Elmer (UK).

### Cell culture

Cells were grown in Dulbecco’s Modified Eagle medium supplemented with 10% foetal bovine serum containing penicillin (100 units/ml)/streptomycin (10 mg/l) and incubated at 37 °C in a humidified atmosphere of 95% air:5% CO_2_. MDA-MB 468 cells and HCT8 were seeded in 96-well micro plates at a density of 30 × 10^3^ cells/ ml for cytotoxic assay and 75 cm^2^ flasks for NMR experiments.

### Cytotoxicity assay

The cytotoxic effect of each drug was assessed by the MTT (3-(4, 5-dimethylthiazol-2-yl)-2, 5- diphenyltetrazolium bromide) assay. Cells were seeded in the 96-well plates and incubated for 24 h at 37 °C in a humidified atmosphere (5%CO_2_: 95% air). The cells were incubated with LY294002 (1.5–100 μM), GSK2334470 (0.3–50 μM), MK2206 (0.1–50 μM), rapamycin (0.1–4000 nM) and AZD8055 (7.5–1000 nM). The IC_50_ values of each drug were calculated using CompuSyn software (ComboSyn, Paramus, NJ) from the absorbance quantitated in a multi-well plate reader with the absorbance at 540 nm.

Drug interactions were calculated with the “combination index (CI)” formula shown below introduced by Chou and Talalay^30^ for the interaction of two drugs D1 and D2:


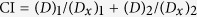


CI < 1, =1, and >1 indicate synergism, additive effect, and antagonism, respectively. The denominator, (D_x_)_1_ and (D_x_)_2_ are the concentrations of each drug alone that inhibits cell growth by x%. The (D)_1_ and (D)_2_ values are the concentrations used in combination that inhibit cell growth by x%.

### Extraction of cellular metabolites

Cells were seeded (2 × 10^6^ cells per flask) in 75 cm^2^ culture flasks until 60–70% confluent, then treated with IC_50_ doses of the respective inhibitors for 24 h. The cells (typically 10^7^ per sample) were then detached by treatment with trypsin and after addition of ice cold media transferred to 1.5 ml microfuge tubes. Briefly, cells were pelleted by centrifuging at 500 g for 5 min at 4 °C and washed twice with ice-cold isotonic saline to remove extracellular phosphate. The metabolites were extracted by a two-solvent system^19^. To the pellets, 20 μl of 1-aminopropylphosphoric acid (0.3 μM) (^31^P-NMR standard), 10 μl of 10 mM EDTA (10 mM) and 0.375 ml of methanol/chloroform (2:1) were added and left on the ice for 1 hour after thorough shaking. Thereafter phase separation was accomplished by adding 0.125 ml of chloroform and Tris-HCl buffer (10 mM) solution (pH 7.4). The mixtures were centrifuged at 1000 g for 10 min at 4 °C. ^31^P-NMR spectroscopy was carried out on the aqueous phase after addition of deuterium oxide (final concentration 10%). A protein assay was carried out, for normalising the NMR data, on the precipitated tissue at the interface of the phases after drying and dissolving overnight in 0.5 ml of NaOH (1 M) and neutralizing with HCl.

### ^31^P NMR spectroscopy

NMR analysis was carried out on a Bruker NMR spectrometer (400 MHz) operating at 161.98 MHz for ^31^P with a ^1^H decouple of 400 MHz and run overnight. The parameters for each acquisition were: acquisition time 1.258 s; relaxation delay 2 s (peak sizes were similar when 1 s or 2 s were used suggesting complete relaxation in 1 s); pulse angle 30°. The ^1^H decouple was maintained throughout the FID acquisition and relaxation delay. Typically 20,000 scans were acquired. All acquisitions were made at 25 °C. At least three independent experiments were carried out and analysed per treatment. Metabolite concentrations were determined by comparison with the standard (0.3 μmoles) resonating at 11.8 ppm^19^.

### Flow cytometry analysis on cell cycle

Cells were seeded in 25 cm^2^ flasks and incubated at 37 °C for 24 h and 48 h then treated with IC_50_ concentrations of each inhibitor for 48 or 24 h then harvested as for the metabolite extraction procedure. After two PBS washes they were re-suspended in 300 μl PBS followed by fixation with 70% ice-cold ethanol during mixing on a vortex. The fixed cells were kept at −20 °C prior to flow cytometry analysis. For the cell cycle analysis, fixed cells were adjusted to 5 × 10^5^ cells/ml and washed 2 times with PBS supplemented with 1% albumin. Then the cells were centrifuged at 1000 g for 5 mins and suspended in 1 ml of staining buffer containing 50 ug/ml propidium iodoide, 50 ug/ml ribonuclease A and 0.1% v/v triton-x-100 in PBS and incubated for 15 min at room temperature. The stained nuclei were kept at 4 °C and protected from light. Flow cytometry was performed using 488 nm laser light on a FACSCalibur flow cytometer (Becton Dickinson) and CELLQuest software (Becton Dickinson) equipped for fluorescence detection, forward, 90° angle light scatter and doublet discrimination. The data analysis was carried out with Flowjo (Or, USA) cell cycling software.

### Western blot analysis of Akt, p-Akt (Ser473), GSKβ and p-GSKβ (ser-9) protein expression

Protein extracts and kinases expression was determined as described previously^10^. MDA-MB 468 cells were treated with the PI3K/Akt/mTOR inhibitors alone or in combination with GSK3 inhibitor SB216763 for 24 h and then washed with 5 ml ice cold PBS and the cells harvested by scraping with 150 μl of lysis buffer (PBS containing 1 mM DTT, a protease inhibitor cocktail, 10 mM sodium pyrophosphate and 1 mM sodium orthovanadate). Samples were kept frozen until they were lysed with a probe sonicator for 30 secs. Protein concentration quantification was carried out with the bicinchonininic acid (BCA) assay. Lysates (25 μg protein/well) were resolved on 4–12% acrylamide Bis–Tris gels (Invitrogen) and the proteins transferred to Immobilon-P polyvinylidene difluoride membranes (Millipore, UK) at 30 V for 1.5 h using a wet transfer system (Invitrogen, UK). Membranes were blocked for 1 h at room temperature in PBS containing 0.1% Tween 20 (PBST), and either 50% sea block for Akt and GSKβ antibodies or 1% (w/v) BSA for p-Akt and p-GSKβ antibodies. Membranes were incubated overnight at 4 °C in PBST containing the appropriate blocking buffer and antibodies at either 1:667 dilution (Akt and p-Akt) or 1:1000 dilution (GSK and pGSK). Membranes were washed four times in PBST prior to 1 h incubation with the appropriate anti-rabbit fluorescent secondary antibody (Licor) in PBST in the dark at (1/10000 dilution). Blots were drained and wrapped in cling film to prevent drying. Blots were imaged using the Odyssey® CLx LI-CORE imaging system to detect fluorescence at 800 nM. Images were then analysed using the Odyssey Image Studio Lite (LI-CORE) software. Each protein ran at the expected molecular weight as previously observed for the Akt and GSK3 proteins. There was only one band on each blot and both the phosphor- and non-phospho version of each protein ran at exactly the same molecular weight. The Akt and pAkt antibodies were used and characterised by our group previously^10^. Full blots for GSK3 and pGSK3 are shown in Supplementary Fig 1 and for Akt and pAKT are shown in Supplementary Fig 2.

### Glycogen synthesis

Cells were seeded (10^6^ per flask) in 25 cm^2^ flasks then treated in triplicate when 70% confluent with LY294002 or AZD8055 or vehicle (DMSO) for 24 h. Glycogen synthesis was determined by a modified version of Coghlan *et al*.^31^. The cells were incubated with 0.25 ml of [^14^C]glucose (55 KBq/ml) for 1 h, washed rapidly with ice-cold PBS, then dissolved in 150 μl of KOH (20%) at 70 °C for 1 h. Glycogen was precipitated by addition of 50 μl of glycogen (2 mg/ml) as carrier and 0.5 ml of ice-cold ethanol and leaving at −20 °C for 2 h. After centrifugation at 10,000 g for 10 min the supernatant was removed and the precipitated glycogen dissolved in 150 ul of hot water, transferred to scintillation vials containing 5 ml of Ultima Gold scintillation fluid (Perkin Elmer UK) and the radioactivity counted in a Tricarb 2100 scintillation counter (Packard UK). The radioactive glycogen value was normalised to protein content carried out on 50 μl of the supernatant.

### Protein assay

Protein assay was done by using the BCA protein assay kit (Sigma-Aldrich UK) with Bovine serum albumin (BSA) as a standard according to manufacturer’s protocol.

### Statistical analysis

All data shown is representative or the average of at least 3 independent experiments. Statistical differences between means were determined using the Student t-test. Significance was set at p < 0.05 and p < 0.01. The IC_50_ values of each drug were calculated using CompuSyn software (ComboSyn, Paramus, NJ) from the absorbance quantitated in a multi-well plate reader with the absorbance at 540 nm.

## Results

### Cell sensitivity to PI3K/Akt/mTOR signalling inhibitors

To investigate the sensitivity of MDA 468 cells to inhibitors of components of the Akt/mTor pathway cells were treated with PI3K, Akt (PKB), mTORC1 and pan-mTORC inhibitors, LY294002, MK2206, rapamycin and AZD8055 respectively. Dose-dependent growth inhibition was seen with all the treatments. The IC_50_ values of each inhibitor on MDA-MB-468 cells is shown in Table 1. The colorectal tumour cell line, HCT8 which over-expresses EGFR, was also treated with LY294002 and AZD8055 and IC_50_ values determined (12 ± 1.9 uM and 83 ± 2.82 nM respectively). Cells prepared for ^31^P-NMR, western blot and flow cytometry analysis in subsequent experiments were treated with IC_50_ drug doses.

### Effect of PI3K/Akt/mTOR signalling inhibitors on expression of pAkt and pGSK3

**Figure 2:**
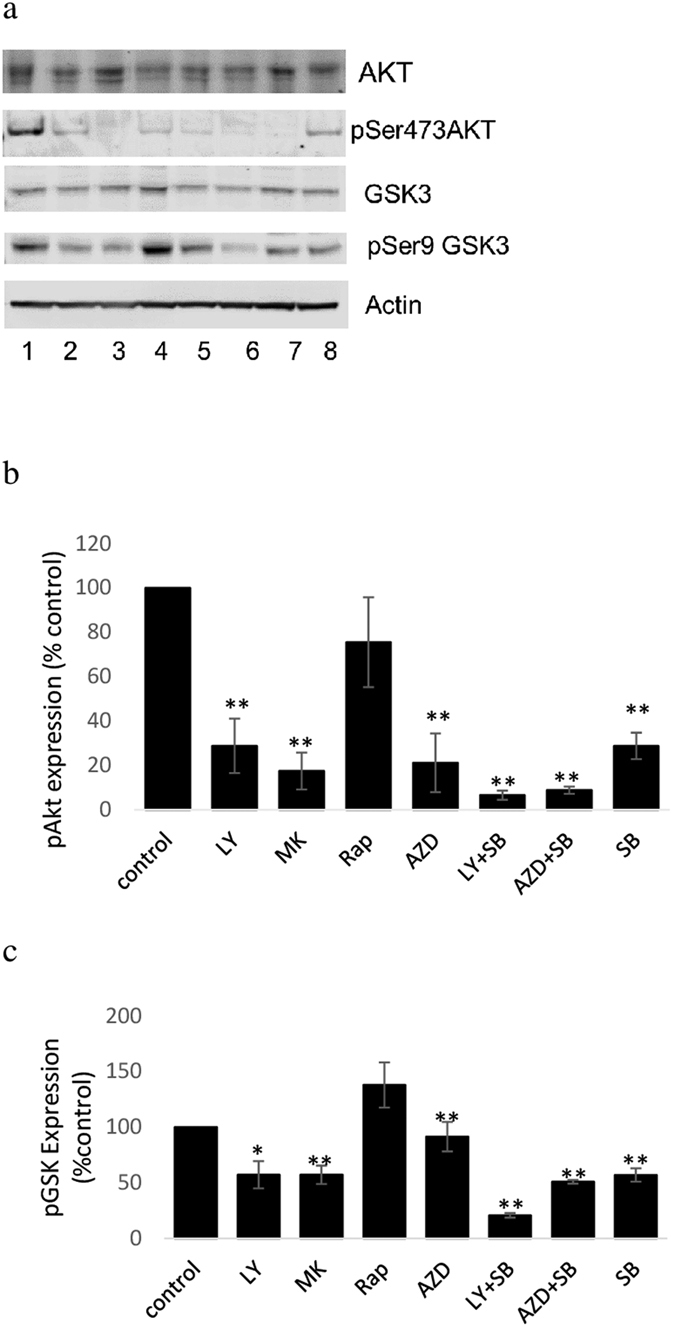
Protein expression of total Akt and GSK3 and their phosphorylation status at Ser473 and Ser9 respectively in lysates of control cells (lane 1), cells treated with LY294002-50 uM (lane 2), MK2206-10 uM (lane3), Rapamycin-20 nM (lane 4), AZD8055-500 nM (lane 5), LY + SB 216367 (lane 6), AZD + SB 216763 (lane 7) and SB 216763 (lane 8) with western blot analysis. Result is representative (**a**) or average ± standard deviation (**b**,**c**) of three independent experiments. For clarity and conciseness only the gel zones with the bands are shown. No other bands were present on the gels than those shown in the figure. All gels were run under the same conditions (described in the methods section). Full blots for GSK3 and pGSK3 are shown in Supplementary Fig 1.

To confirm inhibition of Akt by each inhibitor, protein expression of total Akt and the upstream target of mTOR, Akt-Ser^473^ which is also an important measure of mTORC2 inhibition^32^ was determined using western blotting. In comparison with control samples, treatment with each inhibitor except rapamycin decreased the Akt phosphorylation to very low levels (see Fig. 2). The addition of SB216763 to either LY294002 or AZD8055 further decreased the expression pSer473 over either drug alone. GSK phosphorylation was decreased by treatment of cells with MK2206, confirming the role of Akt in GSK3 phosphorylation. Similarly pGSK3 expression was decreased by treatment with LY294002 or SB216763, but not with rapamycin or AZD8055. The combination of SB216763 with LY294002 significantly decreased pGSK-Ser^9^ expression over either drug alone (LY + SB vs SB t = 5.8, p < 0.01; LY + SB vs LY t = 2.93, p < 0.05). There was no significant difference in the effect of AZD8055 + SB216763 over SB216763 alone.

### PI3K/Akt/mTOR signalling inhibitors and cell cycle distribution

The effect of treatment on MDA-MB-468 cells with LY294002, MK2206, rapamycin and AZD8055 for 24 and 48 h on cell cycle distribution is shown in Table 2. All 4 inhibitors increased cells in G_0_/G_1_ and decreased in S-phase suggesting that they induced G_1_ cell cycle arrest (p < 0.05). In contrast SB216763 treatment decreased cells in G1. There was no detectable subG_1_ peak after any of the treatments. Interestingly the combination of SB216763 with AZD8055 and to a lesser extend LY294002 decreased the build-up of cells in G1 suggesting that SB216763 was antagonising the effect of these two drugs.

### Combination analysis of SB216763 with AZD8055 and LY294002

The apparent antagonising effect of SB216763 on AZD8055 and LY294002 induced G1 blockade contrasts with the findings of enhanced pAkt abrogation with SB216763 and LY294002 and AZD8055. To investigate potential antagonism, MTT assays were carried out on cells treated with SB216763 and either LY294002 or AZD8055 and the data analysed using the Combination Index method of Chou and Talalay^30^. The combination indices are shown in Table 3 and demonstrate that SB216763 antagonises the growth inhibitory effect of AZD8055 and to a lesser extent LY294002 on MDA-MB-468 cells. The antagonistic effect of SB216763 combined with AZD8055 and LY294002 was also demonstrated in HCT8 cells.

### *In vitro*^31^P-NMR spectroscopy of cell extracts

**Figure 3:**
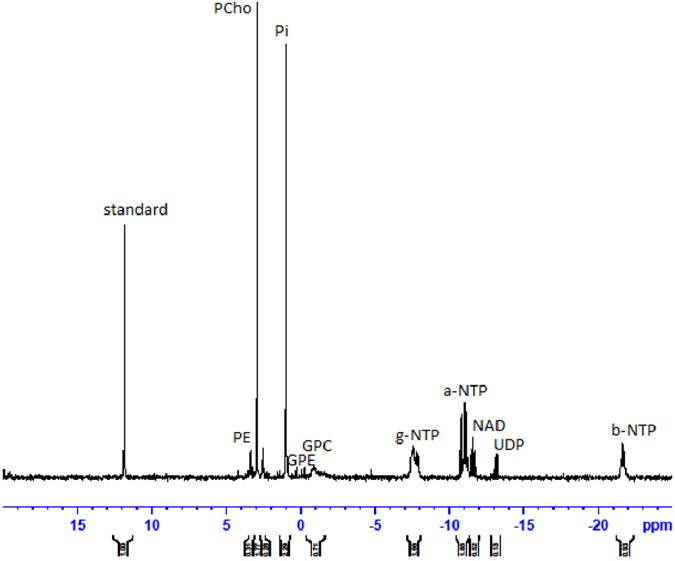
Representative ^31^P-NMR spectrum obtained from the aqueous fraction of control MDA-MB 468 breast cancer cells extract. Phospholipid metabolites are detected according to their chemical shifts (ppm): standard, 1-aminopropylphosphoric acid (0.3 μM); PE, phosphoethanolamine; PC, phosphocholine; Pi, inorganic phosphate; GPE, glycerophosphoethanolamine; GPC, glycerophosphocholine; a-NTP, b-NTP, and g-NTP, nucleotide triphosphates; UDP, UDP-hexose

**Figure 4:**
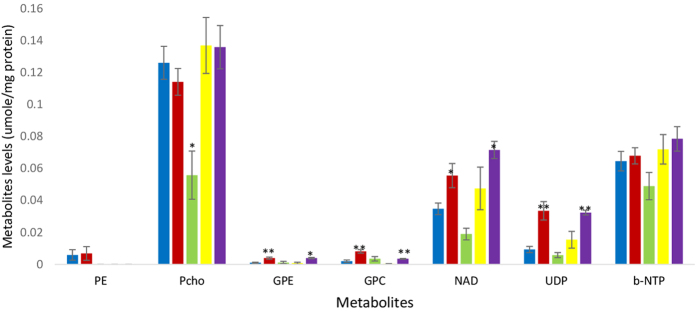
Mean (±SEM) metabolite concentration in cell extracts form MDA-MB-468 cells untreated (blue) or treated for 24 h with LY294002 (red), and MK2206 (green), rapamycin (yellow) and AZD8055 (purple) for 24 h. (units: μmoles/mg protein). Statistically significant difference (*p < 0.05, **p < 0.01) from the control (student t-test).

Figure 3 shows a representative spectrum from an aqueous extract from untreated MDA-MB-468 cells. The phospholipid metabolites evident in the spectrum are PE, PCho, GPE and GPC, UDP-hexoses (uridine-diphosphate-sugar) consisting of UDP-glucose and UDP-N-acetyl-glucosamine (spiked spectra are shown in Supplementary Figs S3 and S4), nucleotide adenine dinucleotide (NAD) and nucleotide triphosphates (NTP) and inorganic phosphorus (Pi), the identities of which were verified by spiking with commercially available compounds. Figure 4 shows the concentration of PCho, GPC, GPE, resonances in the NAD region and UDP-hexoses in cells treated for 24 h with IC_50_ concentrations (metabolite units: μmoles/mg protein) of inhibitors of PI3K, Akt, mTORC1 and mTORC1&2, respectively LY294002, MK2206, rapamycin and AZD8055 which produced inhibitor-specific changes in ^31^P-NMR visible metabolites. PCho content was significantly (t = 2.60, p < 0.05) decreased by treatment with the Akt-specific inhibitor MK2206 but not by LY294002, rapamycin or AZD8055. The content of PDEs, GPC (t = 3.95, p < 0.01) and GPE (t = 4.56, p < 0.01) were significantly increased in cells treated with LY294002 and by treatment with AZD (GPC (t = 4.92, p < 0.01 and GPE t = 4.1, p < 0.05) compared with untreated cells.

Treatment of MDA-MB468 cells with LY294002, but not MK2206, significantly increased the concentration of UDP-hexoses (t = 3.65, p < 0.01) and associated peaks in the NAD region. Treatment of MDA-MB-468 cells with the mTORC1 inhibitor rapamycin did not significantly change the concentration of PCho, GPE, GPC or UDP-hexoses. However in common with LY294002 treatment of cells with the pan-mTORC inhibitor AZD8055 significantly increased the concentration of UDP-hexoses (t = 10.4, p < 0.01).

**Figure 5:**
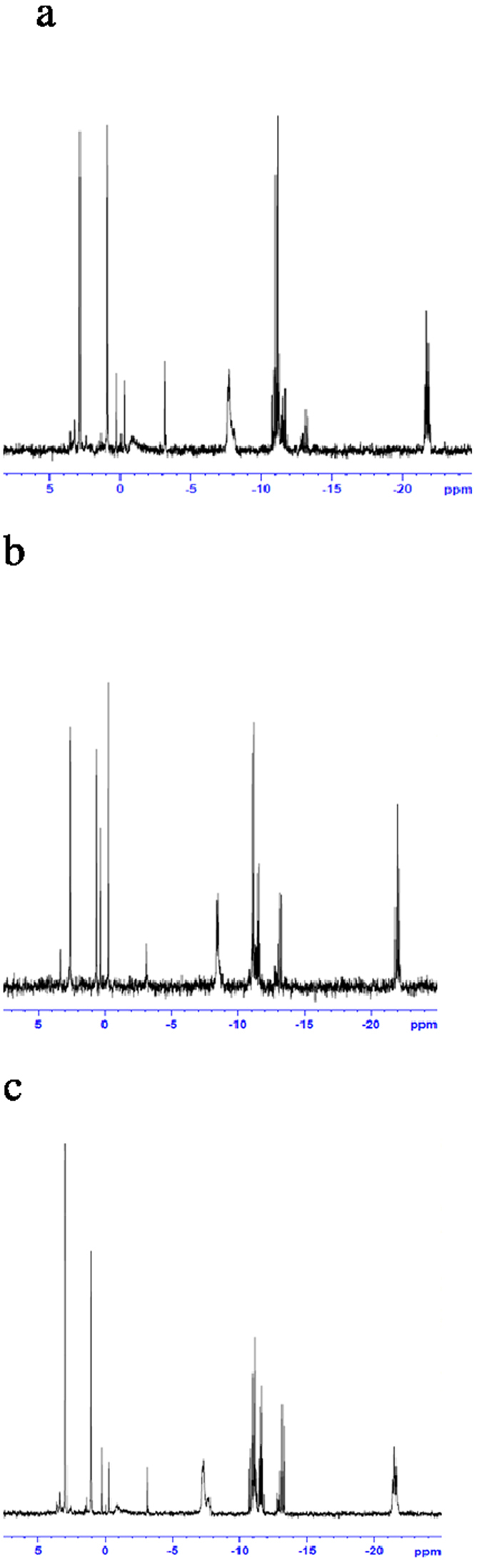
Representative spectra from aqueous phase extracts form untreated (**a**), LY294002 (**b**) AZD8055 (**c**) treated HCT8 cells.

To verify that the increase in UDP-hexoses induced by treatment with LY294002 and AZD8055 was not specific to MDA-MB468 cells the treatments were also carried out on HCT8 cells. In common with MDA-MB-468 cells, treatment of HCT8 cells with either LY294002 or AZD8055 significantly increased the concentration of UDP-hexoses (from 0.02 ± 0.002 to 0.044 ± 0.007, t = 7.4, p < 0.01 and from 0.03 ± 0.013 to 0.066 ± 0.011 t = 3.345, p < 0.02 respectively). Representative spectra from HCT-8 cell extracts are shown in Fig. 5. Treatment of HCT8 cells with LY294002 also increased GPC (t = 3.3, p < 0.05) and GPE content (t = 4.46, p < 0.05).

**Figure 6:**
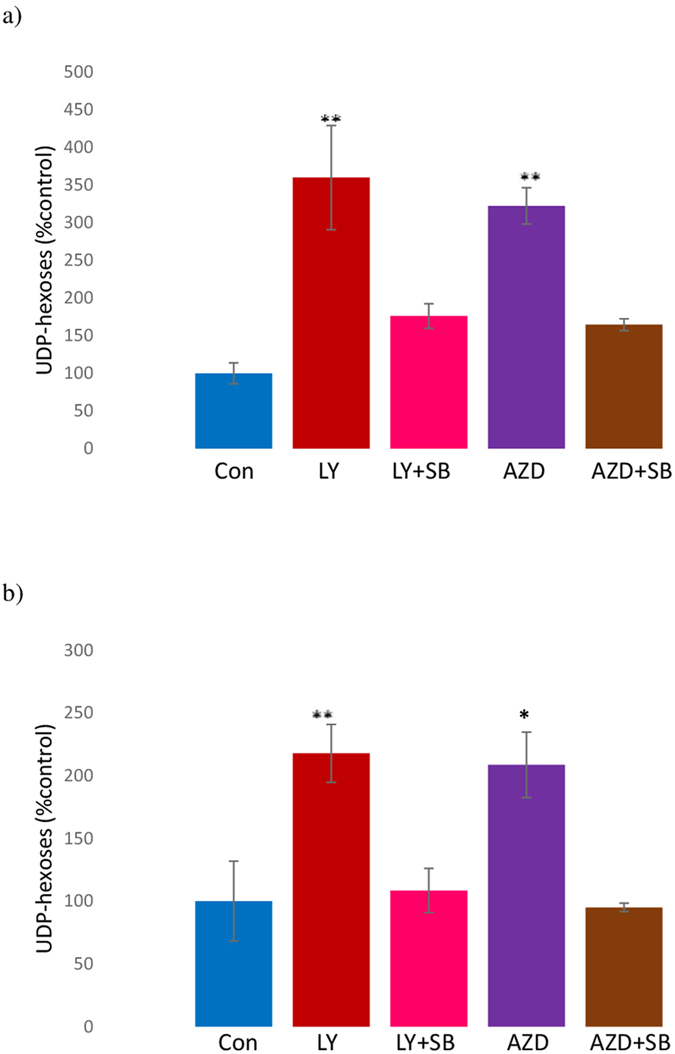
UDP-hexose content (mean ± SEM) in MDA-MB-468 (**a**) and HCT8 (**b**) cells treated for 24 h with LY294002 (LY) (50 μM) (red column), LY294002 (50 μM) and SB216763 (33 μM) (LY + SB) (pink), AZD8055 (500 nM) (AZD) (purple), AZD8055 (500 nM) and SB216763 (33 μM) (AZD + SB) (brown) and untreated (blue). (Normalised to protein content and expressed relative to untreated cells.) Statistically significant difference (*p < 0.05, **p < 0.01) from the control (student t-test).

The UDP-hexoses peak includes UDP-glucose which is an intermediate in the formation of glycogen synthesis, a step catalysed by glycogen synthase (GS). To determine if activation of GSK3, which inhibits GS, is a possible mechanism for the AZD8055 and LY294002 induced increase in UDP-hexoses, MDA-MB-468 and HCT cells were treated with AZD8055 or LY294002 in the presence of the GSK3 inhibitor SB216763 (33 μM). The results from MDA-MB468 and HCT8 cells treated with AZD8055 with SB216763 are shown in Fig. 6. The UDP-hexoses content was significantly less in MDA-MB-468 cells when the SB216763 was co-incubated with cells treated with LY294002 (t = 3.28, p < 0.05) or AZD8055 (t = 8, p < 0.02) compared with LY294002 or AZD8055 alone. Similar results were found with HCT8 cells (Ly vs LY + SB: t = 6.36, p < 0.01) (AZD vs AZD + SB: t = 3.89, p < 0.05). This suggests that LY294002 and AZD8055 are increasing the activity of GSK. Treatment of cells with SB216763 did not affect the concentration of UDP-hexoses compared with untreated cells (For a representative spectrum see Supplementary Fig S5). Mean GPE and GPC content in SB216763-treated cells were significantly (p < 0.05 in each case) different from the control. In contrast to the pan mTORC inhibitor AZD8055, treatment of MDA-MB-468 cells with the mTORC1 inhibitor rapamycin did not increase UDP-hexose concentration suggesting that mTORC2 is important in controlling GSK activity.

**Figure 7:**
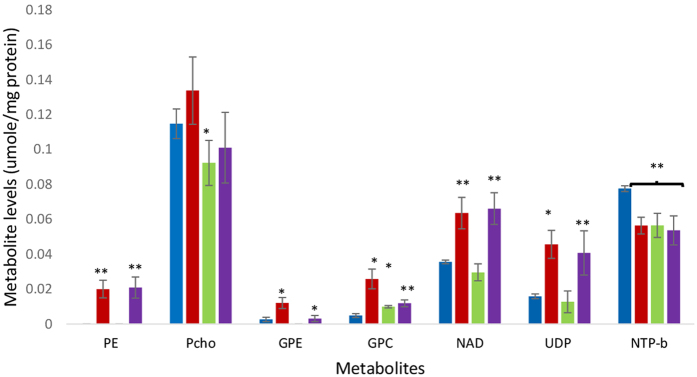
Mean (±SEM) metabolite concentration in cell extracts from MDA-MB-468 cells untreated (blue) or treated for 72 h with LY294002 (red), and MK2206 (green) and AZD8055 (purple) for 72 h. (units: μmoles/mg protein). Statistically significant difference (*p < 0.05, **p < 0.01) from the control (student t-test).

To determine the persistence of the increase in UDP-hexoses induced by treatment with LY294002 and AZD8055, cells were treated for 72 h with each drug. Figure 7 shows that the UDP-hexoses peak is increased in extracts from MDA-MB-468 cells treated for 72 h with LY294002 (t = 2.68, p < 0.05) and AZD8055 (t = 12.1, p < 0.01). The concentration of GPE and GPC are also increased compared with untreated controls after treatment with LY294002 (GPE: t = 3.06, p < 0.05, GPC: t = 2.68, p < 0.05) and AZD8055 (GPE: t = 2.78, P < 0.05, GPC: t = 6.2, P < 0.01). Whilst the concentration of PCho is unchanged by treatment with either AZD8055 or LY294002 but decreased by treatment with MK2206 (t = 2.8, p < 0.05). Treatment with LY294002, MK2206 and AZD8055 for 72 h were found to significantly (t = 3.4 P < 0.05; t = 5.3, p < 0.01; t = 4.5 p < 0.05 respectively) decrease βNTP content.

PI3K regulates Akt via PDK1 which also regulates other targets including PKC and PKA. To determine whether or not inhibition of these targets increases the concentration of UDP-hexoses, MDA-MB-468 cells were treated with IC_50_ concentrations of the PDK1 inhibitor GSK2334470 (5 μM), the PKC inhibitor staurosporine (20 nM) and the PKA inhibitor H-89 (15 μM) for 24 h (representative spectra are shown in Supplementary Figs S6, S7 and S8). UDP-hexose content was not changed in MDA-MB 468 cells treated by either GSK2334470 or staurosporine whilst treatment of cells with the H89 for 24 h completely abolished the UDP-hexoses peak. This corroborates the finding that PKA inversely regulates the formation of UDP-glucose which has been shown to occur via regulation of the expression of UDP-glucose pyrophosphorylase (UGP1)^33^. Decreased PCho content (t = 4, p < 0.05) and pGSK expression (see Supplementary Fig 9) were also apparent after treatment of MDA-MB-468 cells with H-89.

**Figure 8:**
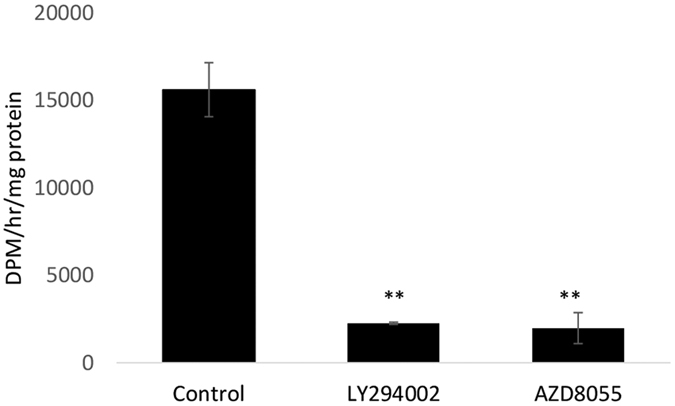
Rate of formation of [^14^C]glycogen in control cells and cells treated for 24 h with Ly294002 or AZD8055 followed by incubation with [^14^C]glucose for 1 h. (units: DPM/h/mg protein). Statistically significant difference (**p < 0.01) from control.

The effect of LY294002 and AZD8055 on glycogen synthesis was measured to confirm that these compounds were influencing glycogen formation. The results in Fig. 8 demonstrate significant inhibition of glycogen synthesis by LY294002 (t = 14.9, p < 0.001) and AZD8055 (t = 13.2, p < 0.001).

## Discussion

The PI3K/Akt/mTOR pathway has many inputs, branching points and effector targets which will likely underlie the disparate changes in phospholipid metabolite content induced by growth inhibitory concentrations of the inhibitors used in this study despite similar effects on cell cycle distribution and Akt inactivation. With the exception of rapamycin each inhibitor decreased phosphorylation of Akt-Ser^473^. Decreased p-Akt-Ser^473^ by treatment with AZD8055 confirmed its inhibition of mTOR2 as p-Akt-Ser^473^ is an important measure of mTOR2 activity inhibition^32^,^34^. In combination of SB216763 (GSK3 inhibitor) with LY294002 or AZD8055 decreased the expression of p-Akt-Ser^473^ over either drug alone. Similarly Fujiki *et al*.^35^ showed that inhibition of GSK3 by SB216763 in combination with LY294002 enhanced the LY294002-induced down-regulation of pAkt in HK-2 renal proximal tubular epithelial cells. Other work^36^ has demonstrated that treatment with SB 216763 for 24 h down-regulates Akt in adrenal chromaffin cells.

The effect of PI3K/Akt/mTOR signalling pathway inhibitors (24 h and 48 h treatment) on cell cycle distribution in MDA-MB 468 cells was assessed using flow cytometry. In common with other studies^24^,^25^, all the PI3K/Akt/mTOR inhibitors increased the number of cells in G_0_G_1_ and decreased the number of cells in S phase suggesting G_1_ cell cycle arrest. However SB216763 decreased the number of cells in G_0_G_1_ and increased the number in G_2_.

UDP-glucose is an intermediate in glycogen synthesis, a step catalysed by glycogen synthase which is inhibited by GSK3. GSK3 itself is inhibited by phosphorylation at Ser^9^ and ser^21^ of GSK3β and GSK3α respectively including by the upstream modulator AKT kinase^37^,^38^. Both the PI3K inhibitor LY294002 and the pan-mTORC inhibitor AZD8055 increased the cellular concentration of UDP-hexoses which was prevented by co-incubation with the GSK-3 inhibitor SB216763. However whilst LY294002 decreased the level of pGSK-3, in agreement with previous work^39^, AZD8055 did not. The specific Akt inhibitor MK2206 also decreased pGSK-3 but did not increase the concentration of UDP-hexoses. Taken together these findings suggest that the appearance of UDP-hexoses peak requires modulation of another pathway besides AKT/mTORC1 and that the level of p-GSK-3 expression does not necessarily affect UDP-hexose content. A recent study has shown that PI3K suppresses the activity of GSK3 by an Akt-independent pathway^16^ and that inhibition with LY294002 activates GSK3.

Glycogen synthase kinase 3 is regulated by several mechanisms^13^ including phosphorylation, protein complexation which control cytoplasmic and organelle GSK3 activity. Thus cell disruption would disturb some or all of the GS regulatory processes so precluding an accurate determination of its activity. Decreased phosphorylation increases its activity but other mechanisms which maybe at the functional level further modulate its activity. As the increase in UDP-hexoses content induced by treatment with LY294002 or AZD8055 can be prevented by the GSK-3 inhibitor SB216763 this would suggest that the activity of GSK-3 is key to UDP-hexoses content. Our study also suggests that GSK3 inactivation mediated by PI3K activation may be more important in regulating glycogen metabolism in cancer cells than Akt. GS activity is readily measurable in intact cells by determination of the rate of conversion of radiolabelled glucose into glycogen. Using this assay we showed that both LY294002 and AZD8055 greatly decreased glycogen synthesis.

Although PI3K regulates Akt via PDK1 which also regulates other targets including PKC, increased UDP-hexoses content was not seen with the treatments of either the PDK1 inhibitor GSK2334470 or the PKC inhibitor staurosporine suggesting that a further pathway between PI3K and GSK3 must be involved. As the pan mTOR inhibitor AZD8055, but not rapamycin treatment of MDA-MB-468 cells increased UDP-hexose concentration this suggests that mTORC2 but not mTORC1 has a role in controlling GSK3 activity but this does not appear to be due to control of the level of phosphorylation of GSK3. Inhibition of PKA, has been shown^33^ to activate glycogen formation and here was found to completely eliminate the detectable UDP-hexose signal corresponding with increased glycogen synthesis from UDP-hexose. Further demonstrating the relationship between GS activity and this signal.

The PI3K/Akt pathway has been reported to progress the cell cycle by up-regulating the cyclin D1^14^ and reducing p27 through inhibition of GSK3 kinase. Whilst the combination of LY294002 or AZD8055 with SB216763 enhanced pAkt inhibition, this did not translate into an increased growth inhibitory effect as both the cell cycle distribution data and MTT assay indicated that these drug combinations were antagonistic. Inclusion of SB216763 (GSK3 inhibitor) in the treatment of either LY294002 or AZD8055 in both MDA-MB-468 and HCT 8 cells, prevented the increase in UDP-hexoses peak supportive of its potential role as a response marker. The increased level of the uridine-5′-diphosphate sugars, UDP-glucose and UDP-N-acetyl-glucosamine after 24 h treated with LY294002 and AZD 8055 in both MDA-MB 468 breast cancer was also observed after 72 h of treatment suggesting that it may be a stable imaging biomarker of response to these drugs.

Glycolysis is known to be decreased by inhibition of PI3K^10^ which may increase the availability of glucose for other metabolic pathways that are dependent on glucose including the hexosamine pathway. Thus whilst changes in glycogen formation induced by modulation of GSK3 activity may influence UDP-glucose levels parallel effects on UDP-N-acetylglycosamine formation may occur due to increased availability of cellular glucose.

Changes in PME levels detected by Phosphorus-NMR spectroscopy has been shown to predict cancer response to chemotherapy^18^,^40^ and a number of studies have suggested that the PME component, PCho, the concentration of which has been shown to be associated with proliferation^41^,^42^, may be a marker of response to cell-signalling inhibitors including those targeting the Akt pathway^24^,^25^,^26^,^27^,^43^,^44^,^45^. However whilst some studies have demonstrated decreased PCho content associated with response^24^,^25^ others have shown increased^26^,^27^ or no significant change^45^ in PCho accompanying response to treatment with drugs targeting PI3K/Akt/mTOR.

Al-saffar *et al*.^24^ showed that treatment of prostate cancer cells with the PI3K inhibitor, PI-103 which caused G_1_ cell cycle arrest and decreased Akt phosphorylation was accompanied by decreased PCho concentration. Beloueche-Babari *et al*.^25^ treated MDA-MB231, MCF7 and Hs578T cells with LY294002 for periods ranging from 24–40 h depending on the drug and the cell type detected significant decrease in PCho content compared with untreated cells. The variability in PCho is illustrated by PCho content of MDA-MB231 cells treated with LY294002 for 40 h did not show a significant drop compared with control cells^43^. Further Moestue *et al*.^26^ showed that a 3 day treatment of xenografts representing basal-like breast cancer with MK2206 or the dual PI3K/mTOR inhibitor BEZ235, which decreased pAkt expression significantly increased PCho levels. Our results demonstrated a lack of consistency in the effect of PI3K/Akt/mTOR pathway inhibitors on PCho content of cancer cells, suggesting that this metabolite is not a robust response marker. Variation in the changes in PCho level was observed between the different drugs. Thus PCho content is not changed by the 24 h treatments with the inhibitors of PI3K or PDK1 but treatment with specific Akt inhibitor MK2206 caused a significant decrease in PCho. This is in agreement with other work which has shown that inhibitors of Akt^46^ decrease PCho specifically due to decreased Choline Kinase activity. However both LY294002 and AZD8055 also decrease pAkt expression but do not decrease PCho content which may be due to their additional effects on other kinases.

The phosphodiesters GPE and GPC were found to be increased by treatment of cells with the PI3K inhibitor, LY294002. Moestue *et al*. 2013^47^ also demonstrated increased GPC relative to controls in breast cancer cells treated with BEZ235 associated with decreased proliferation and pAkt-ser473. However, here cells treated with the Akt inhibitor MK2206 did not increase PDE content but cells treated with the pan-mTOR inhibitor did. Accumulation of GPE and GPC cells treated with LY204002 could be due to the inhibition of the metabolism of lipid breakdown products causing accumulation of PDEs by PLC, PLD and PLAs or decrease in production of second messengers for the signalling pathways activation such as diacylglycerol (DAG) and phosphatidic acid (PA) from the Kennedy pathway^44^,^48^. However the complex role of GPC in cancer^47^ is very difficult to understand and elucidating the mechanism of therapy-induced changes in GPC is likely to be challenging.

In summary, inhibition of components of the PI3K/Akt/GSK3/mTOR in EGFR-overexpressing MDA-MB 468 breast cancer and HCT8 colon cancer cell lines is associated with inhibitor-specific changes in PCho content. Whilst, activation of GSK3 downstream of PI3K and mTOR inhibition indicates that there might be a regulatory loop between the GSK3 and PI3K or mTOR2 kinases resulting in increased levels of UDP-hexoses, which are compounds visible in *in-vivo* 31P-NMR spectra^49^. UDP-hexoses were consistently increased by treatment with PI3K and pan-mTOR inhibitors (LY294002 and AZD8055 respectively) and may be an early response biomarker to those inhibitors.

## Additional Information

**How to cite this article**: Phyu, S. M. *et al*. Probing the PI3K/Akt/mTor pathway using ^31^P-NMR spectroscopy: routes to glycogen synthase kinase 3. *Sci. Rep.* **6**, 36544; doi: 10.1038/srep36544 (2016).

**Publisher’s note:** Springer Nature remains neutral with regard to jurisdictional claims in published maps and institutional affiliations.

## Supplementary Material

Supplementary Information

## Figures and Tables

**Table 1:** IC_50_ s of each signalling inhibitor on MDA-MB-468 cells treated for 72 h measured by MTT (3-(4, 5-dimethylthiazol-2-yl)-2, 5- diphenyltetrazolium bromide) assay.

Inhibitors	Targets	IC_50_
LY294002	PI3K	23.7 ± 12.2 uM
GSK 2334470	PDK1	5.2 ± 2.8 uM
MK2206	pan Akt	2.1 ± 0.2 uM
Rapamycin	mTOR	48.6 ± 7.1 nM
AZD 8055	mTOR1/2	65.0 ± 11.0 nM
SB 216763	GSK3	38.5 ± 3.9 uM

The IC_50_ values of each drug were calculated using CompuSyn software (ComboSyn, Paramus, NJ).

**Table 2:** Effect of inhibition of PI3K/Akt/mTOR signalling pathway inhibitors on cell cycle distribution for both 24 and 48 h with flow cytometry analysis. DNA analysis shows % increase in G1 and decrease in S and G2 fractions in various treated samples (mono and combination therapies) compared with control untreated samples in MDA-MB 468 cells proving cell cycle arrest (LY: LY294002, AZD:AZD8055, SB: SB216763).

	24 h treatment			48 h treatment		
	%G1	%S	%G2/M	%G1	%S	%G2/M
Control	44.1 ± 5.7	19.0 ± 2.8	34.5 ± 13.4	51.4 ± 14.5	9.8 ± 1.4	36.7 ± 12.7
LY294002	80.8 ± 1.8	2.5 ± 0.7	16.2 ± 3.1	81.2 ± 3.4	5.7 ± 0.7	11.7 ± 3.4
MK2206	75.8 ± 4.5	10.7 ± 6.6	12.6 ± 2.0	75.0 ± 4.2	7.0 ± 1.4	16.0 ± 1.4
Rapamycin	78.0 ± 1.4	6.5 ± 0.7	14.3 ± 1.8	68.3 ± 4.9	8.7 ± 0.6	22.0 ± 7.0
AZD8055	77.0 ± 2.8	6.0 ± 0.0	15.5 ± 3.5	77.3 ± 5.0	4.7 ± 0.6	16.3 ± 4.5
LY + SB	69.0 ± 4.2	4.0 ± 0.0	24.0 ± 2.8	71.7 ± 2.1	5.8 ± 2.5	20.6 ± 3.1
AZD + SB	56.0 ± 0.0	13.0 ± 0.0	28.0 ± 0.0	54.0 ± 14.1	9.5 ± 3.5	33.5 ± 10.6
SB				38.9 ± 1.9	9.7 ± 3.6	56.0 ± 2.1

**Table 3:** Combination indices (CI) for LY and AZD with SB216763. CI = (D)1/(Dx)1 + (D)2/(Dx)2 If CI < 1, =1, and >1 indicate synergism, additive effect, and antagonism, respectively.

Combination Index (CI)	MDA-MB 468	HCT 8
LY294002 + SB216763	1.139 ± 0.085	2.3 ± 0.3
AZD8055 + SB216763	2.113 ± 0.54	1.48 ± 0.21

The denominator, (Dx)1 and (Dx) 2 are the concentrations of each drug alone that inhibits cell growth by x%. The (D)1 and (D)2 values are the concentrations used in combination that inhibit cell growth by x%.

**Figure 1:**
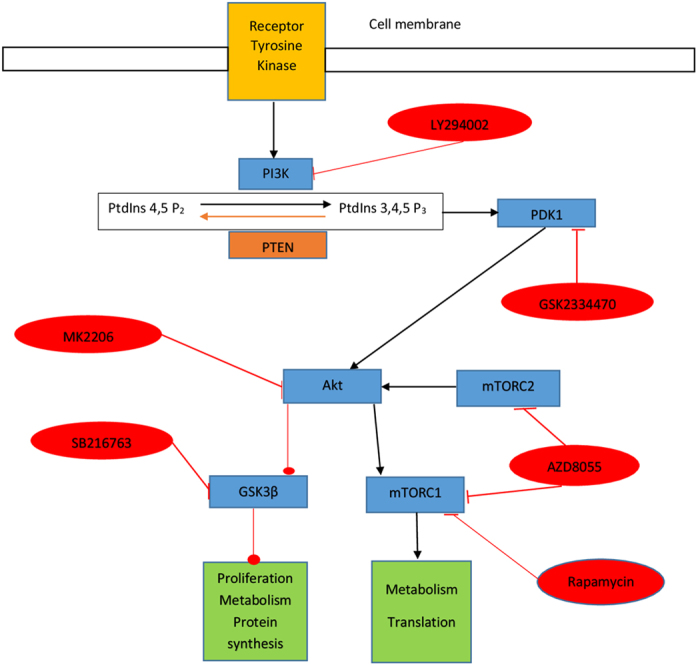
Key components of the PI3K/Akt/mTOR signalling pathway showing activator (black arrows) and inhibitory associations (red lines) and commercially available inhibitors. Abbreviations: mTORC mechanistic target of rapamycin complex; PI3K phosphatidylinositol kinase; PtdIno Phosphatidylinositol-4,5-bisphosphate 3-kinase; PDK1 Phosphoinositide-dependent kinase-1; PTEN phosphatase and tensin homolog.
