# Supplementary material for: Probing the PI3K/Akt/mTor pathway using 31P-NMR spectroscopy: routes to glycogen synthase kinase 3
Source: Sci Rep. 2016 Nov 4;6:36544. doi: 10.1038/srep36544 (PMC5109916; doi:10.1038/srep36544)

**Probing the PI3K/Akt/mTor pathway using 31P-NMR spectroscopy: routes to glycogen synthase kinase 3**

**Su M Phyu, Chih-Chung Tseng, Ian N Fleming, Tim AD Smith***

**Supplementary Data File**

Supplementary Fig 1: GSK and pGSK western blots showing entire blot

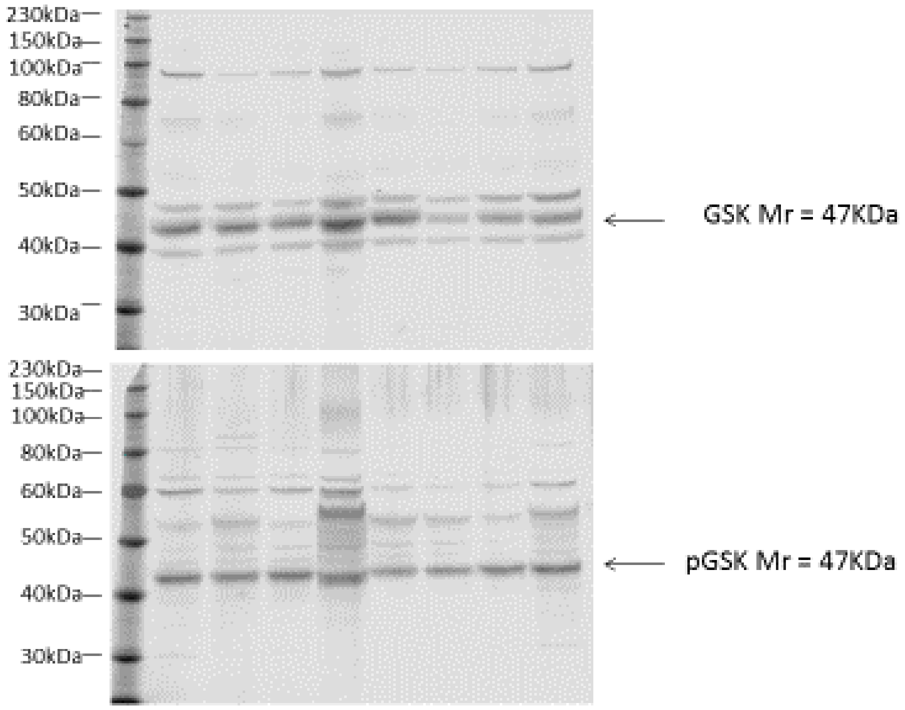


| 1 | 2 | 3 | 4 | 5 | 6 | 7 | 8 | 9 |
|---|---|---|---|---|---|---|---|---|

Molecular weight markers (lane 1) lysates of control cells (lane 2), cells treated with LY294002-50uM (lane 3), MK2206-10uM (lane4), Rapamycin-20nM (lane 5), AZD8055-500nM (lane 6), LY+SB 216367 (lane 7), AZD+SB 216763 (lane 8) and SB 216763 (lane 9).

Supplementary Fig 2 – AKT and pAKT western blots showing entire blot

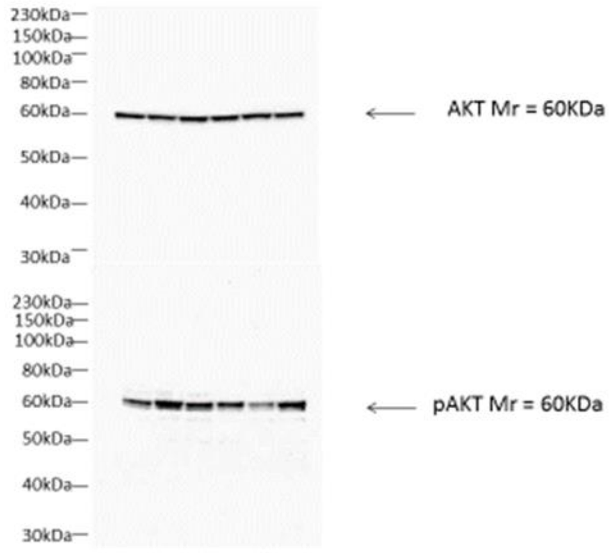

Supplementary Fig S3: $^{31}$P-NMR spectra of AZD8055 treated cells extract before (Red) and after (blue) addition of commercially available UDP-glucose. Whole spectra (A) and Expanded (B).

A

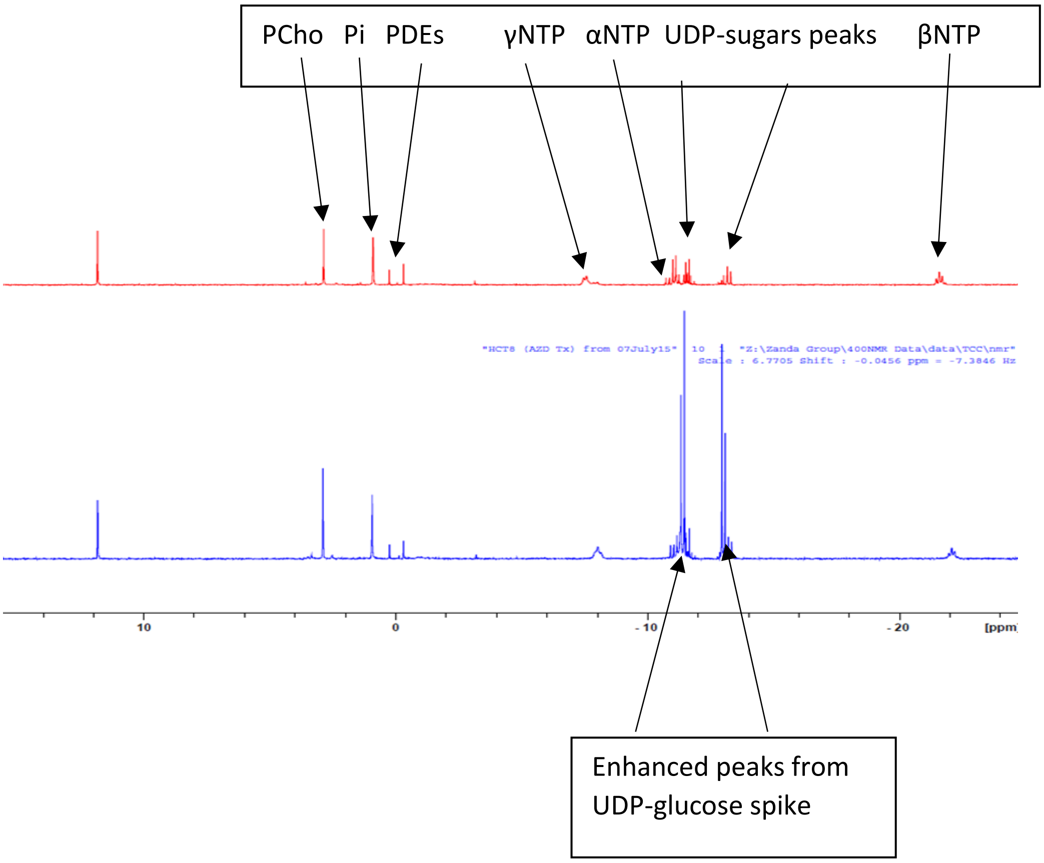


B

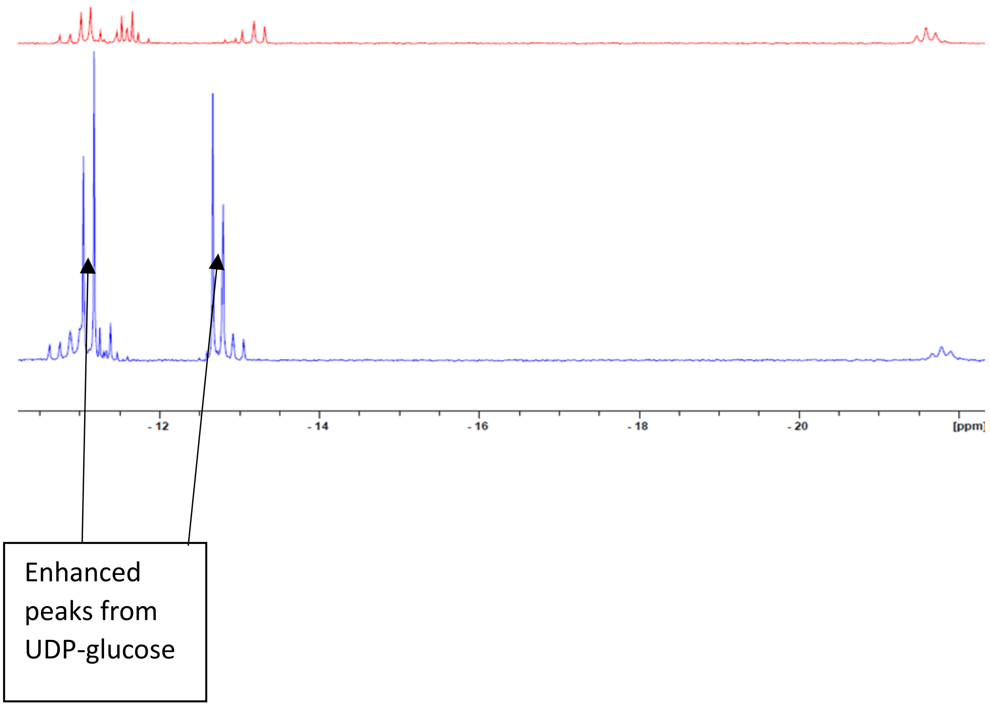

Supplementary Fig S4: $^{31}$P-NMR spectra of LY294002 treated cells extract before (blue) and after (red) addition of commercially available UDP-Nacetylglucosamine. Whole spectra (A) and expanded (B).

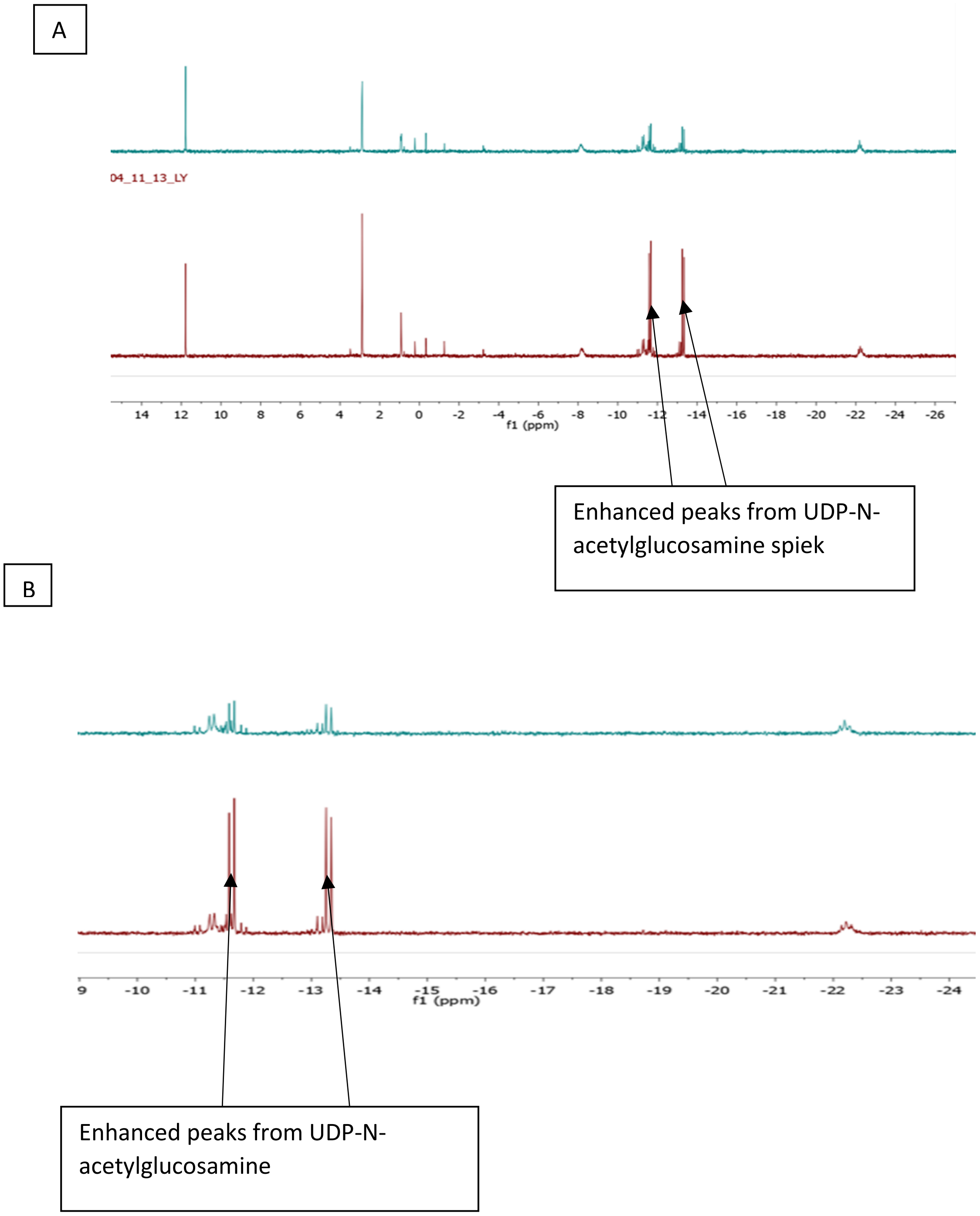

Supplementary Fig S5: Representative spectra of extracts from control (A) and SB216763 (33µM) treated (B) MDA-MB-468 cells

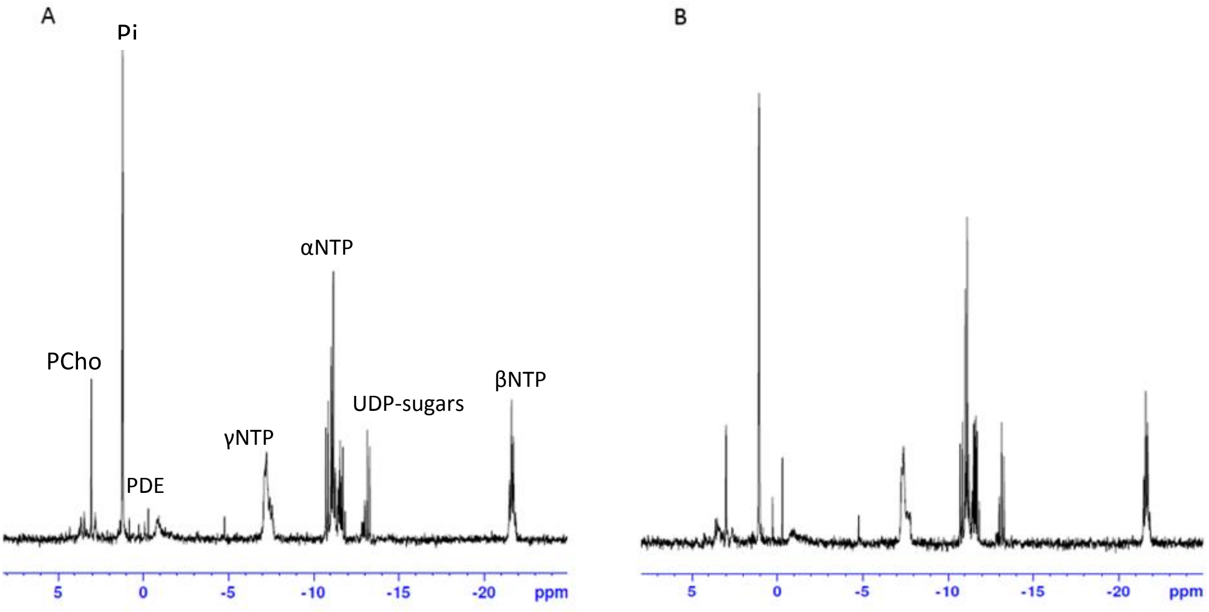


Supplementary Fig S6) Representative spectra of extracts from control (A) and staurosporine treated (B) MDA-MB-468 cells

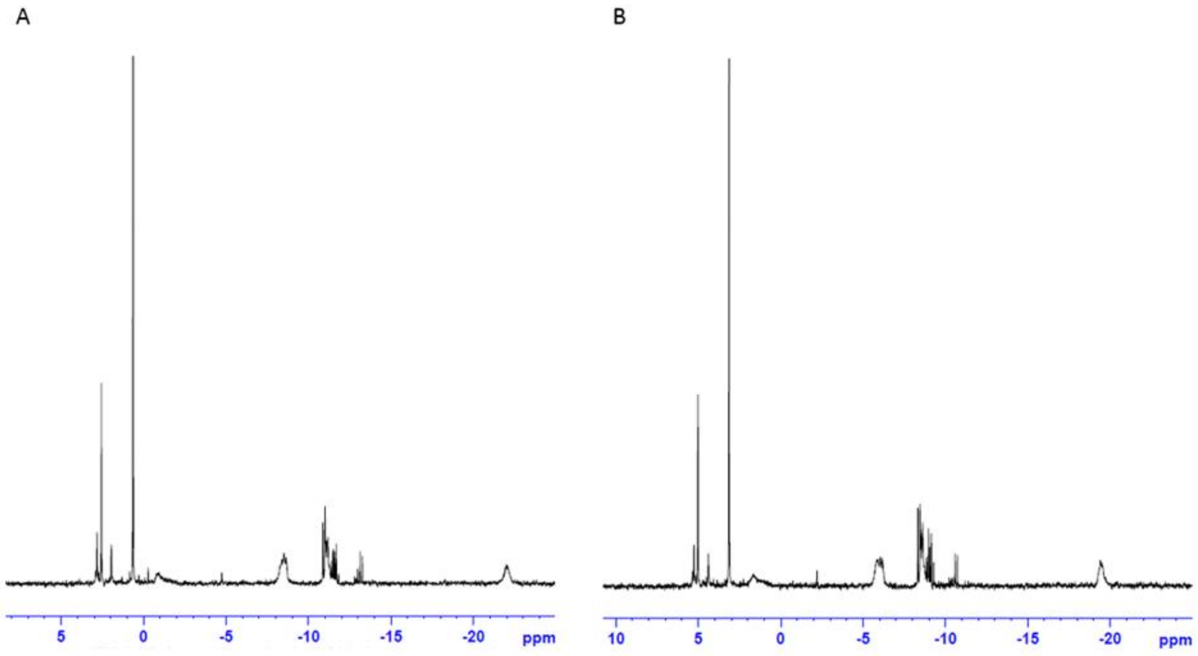

Supplementary Fig S7: Representative spectra of extracts from control (A) and GSK2334470 (B) treated MDA-MB-468 cells

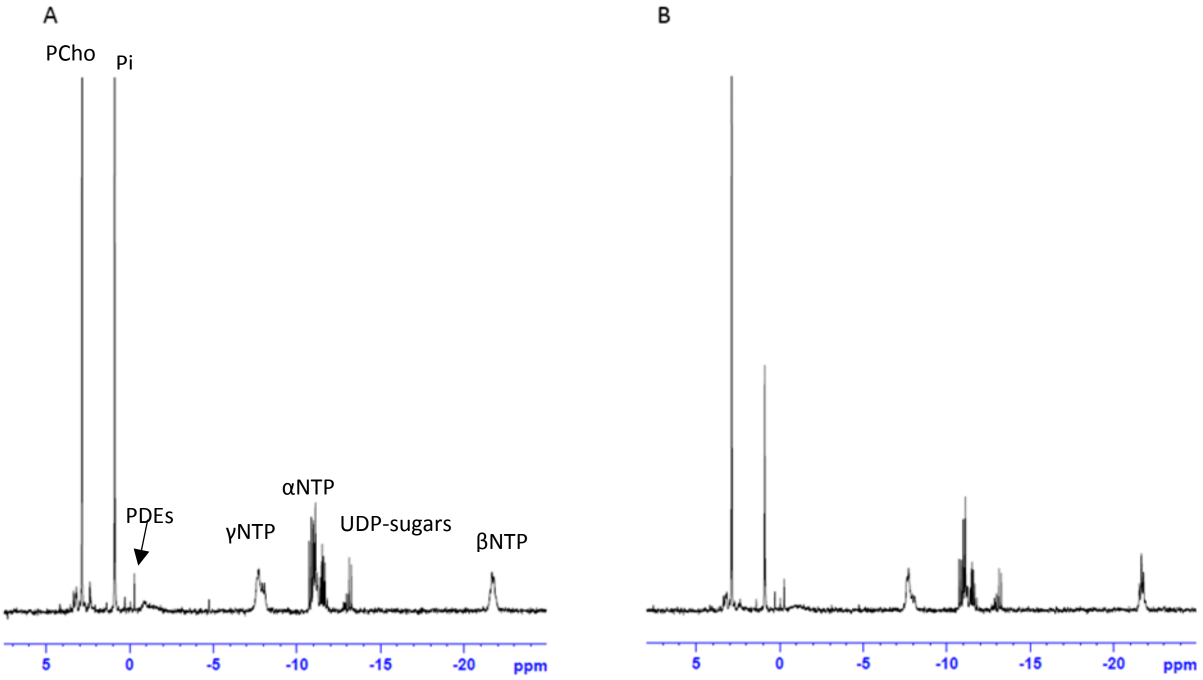


Supplementary Fig S8: Representative spectra of extracts from control (A) and H-89 treated (B) MDA-MB-468 cells

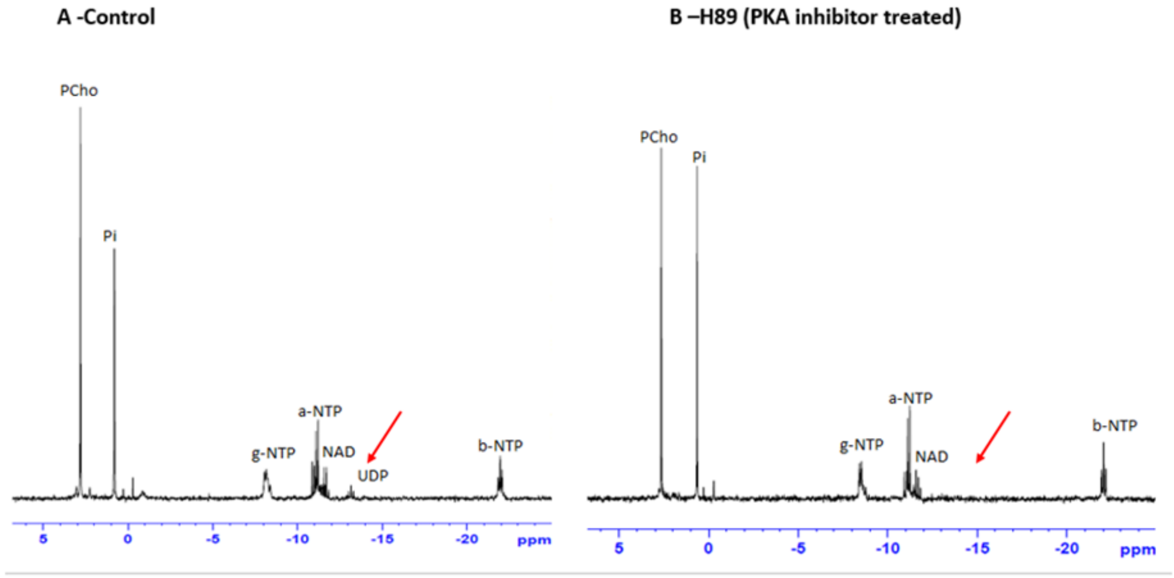

Supplementary Fig S9) Representative western blot of total and phosphor-GSK in untreated (con) and H89-treated (24h) MDA-MB-468 cells

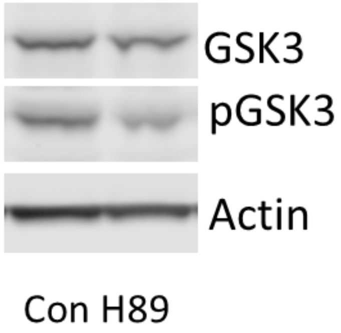

Supplement: Supplementary Information [file srep36544-s1.pdf]
